# Mucosal-Associated Invariant T (MAIT) Cells Are Impaired in Th17 Associated Primary and Secondary Immunodeficiencies

**DOI:** 10.1371/journal.pone.0155059

**Published:** 2016-05-11

**Authors:** Yifang Gao, William Rae, Keseva Ananth Ramakrishnan, Gabriela Barcenas-Morales, Rainer Döffinger, Efrem Eren, Saul N. Faust, Christian H. Ottensmeier, Anthony P. Williams

**Affiliations:** 1 Academic Unit of Cancer Sciences, Faculty of Medicine, University of Southampton and Southampton NIHR Experimental Cancer Medicine Centre, Southampton, United Kingdom; 2 Department of Clinical Immunology, Addenbrookes Hospital, Cambridge, United Kingdom; 3 NIHR Wellcome Trust Clinical Research Facility, University Hospital Southampton NHS Foundation Trust, Southampton, United Kingdom; 4 Department of Clinical Immunology, University Hospital Southampton NHS Foundation Trust, Southampton, United Kingdom; Karolinska Institutet, SWEDEN

## Abstract

The recently described Mucosal Associated Invariant T (MAIT) cells mediate specific recognition of bacterial and fungal vitamin B2 metabolites. As innate T cells, they possess broad effector responses, including IFN- including Iproduction, that are comparable to conventional T cell responses. Immunodeficiencies associated with systemic Th17 deficiency may also be compounded by defects in MAIT immunity. We evaluated Th17 immunity in this innate T cell compartment in primary (AD-HIES) and secondary immunodeficiency (thymoma) patients with conventional Th17 deficiency and susceptibility to fungal and bacterial disease. Our results suggest that MAIT cells are both reduced and functional deficient in STAT3 deficiency and thymoma patients with IL-12/23 autoantibodies. In contrast, thymoma patients without autoantibodies preserved the normal number and functional MAIT cells.

## Introduction

The factors that contribute to an increased susceptibility to fungal infection have been illuminated through the study of patients with primary and secondary immunodeficiencies [1, 2]. These studies have evaluated individuals with primary immunodeficiencies such as autosomal dominant STAT3 deficient Hyper IgE Syndrome (AD-HIES) and autosomal dominant STAT1 gain of function Chronic Mucocutaneous Candidiasis (AD-CMC) [3, 4], demonstrating a significant impairment of systemic Th17 immunity [5, 6]. Th17 cells are typically αβ^+^, CD4^+^ polyclonal T cells that are able to produce the key effector cytokines IL-17A/F and IL-22. These cytokines amplify mucosal responses through an action upon epithelial cells that produce important neutrophil chemotactic factors and antimicrobial peptides [7]. Indeed, other primary immunodeficiencies that are selective for the absence of IL17F (autosomal dominant IL-17F deficiency) or lack a response to IL17 (autosomal recessive IL-17RA deficiency) further illustrate the importance of IL-17 production for control of bacterial (i.e. *S*. *aureus)* and fungal (i.e. *C*. *albicans)* infection [8]. The generation and maintenance of Th17 cells has been shown to be dependent on the cytokines IL-6, IL-23 and IL-1β. Certain secondary immunodeficiencies that can mimic aspects of HIES and CMC have been shown to have inhibitory autoantibodies to these key cytokines [9]. In particular, some thymoma patients have been noted to have biologically active antagonistic autoantibodies to the common IL-12/23 p40 subunit and to IL-17F, leading to Th17 deficiency [10–12].

In recent years, another important IL-17 producing subset of αβ T cells has been identified, namely Mucosal Associated Invariant T cells (MAITs) [13, 14]. MAIT cells are an example of an ‘innate T cell’ [15–17]. These T cells reside principally in tissues but can be identified in peripheral blood [18]. MAIT cells possess a semi-invariant T cell receptor which utilizes Vα7.2-Jα33, with a restricted use of certain Vβ family members (i.e. Vβ2 and Vβ13). These cells are CD161^++^, CD8^+^ or double negative, effector memory cells with chemokine receptor expression that directs tissue tropism [19]. Their antigen recognition is unique in being able to respond to bacterial and fungal vitamin B2 metabolites through presentation on MR-1 [20] or via indirect activation through IL-12 and IL-18 cytokines that act on constitutively expressed IL-12 and IL-18 receptors [21]. We evaluated the presence and function of these cells in two groups of well-characterized primary (HIES) and secondary immunodeficiency (thymoma) patients with conventional Th17 deficiency and susceptibility to fungal and bacterial disease.

## Methods

### Subjects and sample preparation

PBMC from 6 thymoma patients with autoantibodies to IL-12/23 p40 (thymoma positive), 4 thymoma patients without autoantibodies (thymoma negative), three HIES adults with confirmed STAT3 deficiency (V637M, R417S, G618D) and 16 adult controls were isolated using Ficoll (GE Health, UK) according to manufacturer protocol. All participants were free from infection at the time of analysis. Written Informed consents were obtained from all patients (REC13/2000 and REC09/H0502/4). Control samples were obtained from the National Blood Services UK. The study was carried out in accordance with the Declaration of Helsinki. The study was approved by the University of Southampton, School of Medicine ethics committee. (REC13/2000 and REC09/H0502/4).

### Immunophenotyping

PBMC were stained with the following antibodies: TCRC Pacific Blue, CD4 V500, V follow (Biolegend, UK), MR1 APC (26.5) (Biolegend, UK), CD3 PerCP, CD161 APC, CD8 APC-Cy7, CD19 APC-Cy7 and CD20 Pacific Blue. All antibodies were from BD biosciences unless specified. The samples were stained for 15 minutes at room temperature, processed and then analyzed on a FACS Canto II machine with FACS Diva software evaluation.

### IL-17 and IFN-γ production by MAIT and conventional T cells

PMA (Sigma) and ionomycin (Sigma) were used for T cell activation. Briefly, PMA and ionomycin were incubated with PBMC for 6 hours at 37°C, GolgiStop was added after 1 hours of incubation. Cells were fixed and permeabilised by the BD Cytofix/Cytoperm Fixation/Permeabilisation Kit according to the manufacturer’s instruction before staining with the phenotype marker together with IL-17A FITC (eBiosciences) and IFN-γ PE-Cy7 (BD Biosciences). Samples were analyzed on a FACS Canto II machine with FACS Diva software evaluation.

### MAIT cell cytokine assessment

Enriched MAIT cells population were sorted with affymetrix MagniSort system with Vα7.2 antibody (Purity>98%). IL-12 (50ng/ml) (Peprotech, UK) and IL18 (50ng/ml) (Peprotech, UK) were used to stimulate sorted MAIT across the clinical cohorts and controls for 24 hours. The supernatants were then collected and analysed with a cytokine human 25-plex (GM-CSF, TNF-n 25–1β, IL-4, IL-6, MIP-1α, IL-8, IL-15, IFN-α, IL-2R, IP-10, MIP-1β, Eotaxin, RANTES, MIG, IL-12(p40/p70), IL-1RA, IFN-γ, IL-13, MCP-1, IL-7, IL-17, IL-10, IL-5, IL-2) on the Luminex system. 10,000 cells were added to each samples and cytokine values were normalised to the number of MAIT cells (Vα7.2+CD161++CD3+ cells/ Vα7.2+ve cells pre sort) present in this population.

### Statistical analysis

Kruskal–Wallis test were used for the statistical comparison across the groups.

## Results and Discussion

**Fig 1:**
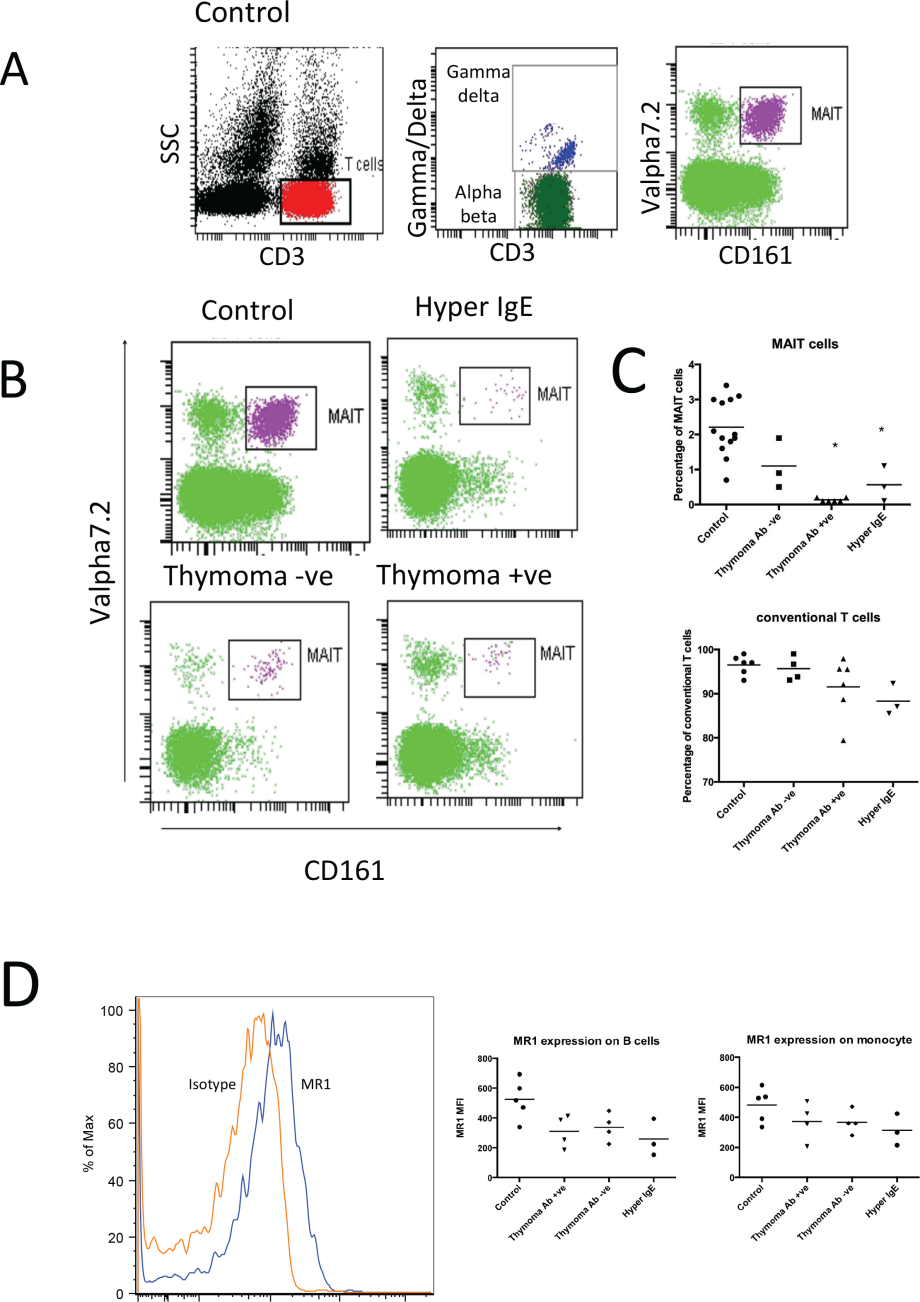
(A) MAIT identification strategy in a healthy control. MAIT cells were identified following sequential gating of CD3 and TCR αβ expression, prior to their Vα7.2 and CD161 co staining. (B) Representative MAIT enumeration from primary and secondary immunodeficiency groups. (C) Mean percentage of MAIT cells (Percentage of MAIT cells in αβ T cells) and conventional T cells (Percentage of conventional T cells in total T cells) present in controls (n = 16, range 1.3–3%), thymoma negative (n = 4, range 0.4–1.9%), thymoma positive (n = 6, range 0.1–0.2%), and HIES (n = 3, range 0.1–1.1%) patients. The percentages of MAIT cells were found to be significantly lower in thymoma positive and HIES patients compared to the healthy controls (*P<0.0001). (D) Representative MR1 expression on control B cell population. Level of MR1 expression on B cell population was not significantly different across the clinical groups.

We identified MAIT cells through a sequential gating strategy that selected CD3+ T cells, conventional αβ T cells and finally Vα7.2/CD161^++^ cells. Many of these MAIT cells had a characteristic CD8αα phenotype. Healthy volunteers showed a distinctive cluster of Vα7.2/CD161^++^ MAIT cells (Fig 1A). Representative enumeration of MAIT cells from the clinical groups showed a notable reduction of MAIT cells in HIES and thymoma +ve patients (Fig 1B). We extended this phenotyping to our cohorts and found that the extent of MAIT deficiency were similar in the thymoma positive patients to that observed in HIES (Fig 1C). The mean percentage of MAIT cells in HIES was 0.57% (P<0.005), and in the thymoma positive patients was 0.13% (P< 0.0001) compared to 2.2% in healthy controls. In contrast, the MAIT population of thymoma negative patients was not significantly different from controls (0.93% vs 2.2%, p = 0.2). The MR1 expression on B cells and monocyte across the clinical groups was not significantly different to controls (Fig 1D).

**Fig 2:**
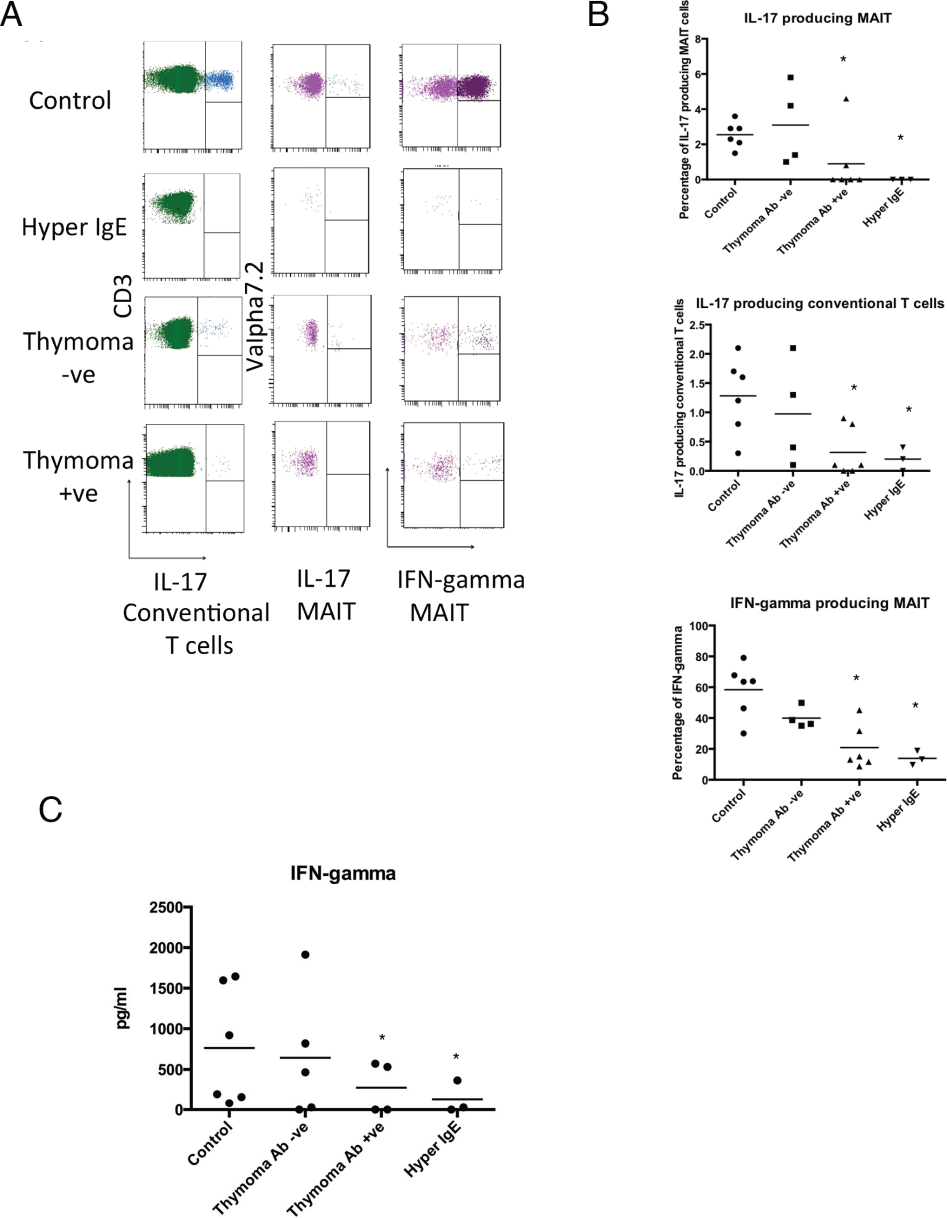
(A) Intracellular cytokine production of Il-17 and IFN-γ by MAIT and conventional T cells in representative control and patient groups. (B) Mean percentage of IL-17 and IFN-γ T cells (MAIT and conventional) in controls and patient groups. The percentage of IL-17 conventional and MAIT cells was significantly reduced in the thymoma positive, HIES patients (*P<0.03). MAIT IFN-γ production was lower in HIES and thymoma positive patients. (C) IFN-γ assessment of enriched MAIT cells across the clinical cohorts following IL-12/18 stimulation.

In agreement with previous studies [8, 12, 22], HIES patients showed a profoundly reduced number of conventional Th17 cells (Fig 2A and 2B) (0.2% vs 1.28%, P<0.03). Within the thymoma cohorts a significant reduction in the Th17 subset was only seen in those with IL-12/23 autoantibodies (Fig 2A and 2B) (0.3% vs 1.28%, P<0.01). Similarly both HIES and thymoma positive patients showed significant reductions in IL-17A producing MAIT cell populations (Fig 2A and 2B). This reduction of IL-17A production from this unconventional T cell subset led to a more extensive IL-17A T cell deficiency in both HIES and thymoma positive patients (Fig 2B).

In agreement with a previous study [19], MAIT cells did not proliferate following mitogen (PMA), TCR (anti-CD3/CD28) or specific innate signaling with IL-12/18 in patients and controls (S1 Fig). Recent studies by several groups have shown that MAIT cells can proliferate under bacteria stimulation. {Leeansyah, 2014 #5;Kurioka, 2015 #6;Leeansyah, 2015 #7}. However, the cells used for these experiments were either pre-activated MAIT cells or fetal MAIT cells. We examined the cytokine production of Vα7.2+ cells through a novel approach involving the activation of enriched MAIT cells within the Vα7.2 population with the specific stimulation of IL-12/18 (S3 Fig). The majority of IFN-gamma production was identified in the CD161+Vα7.2+ MAIT cells. (Other cytokines were not evaluated). In agreement of the intracellular cytokine analysis, a reduction in IFN-γproduction was found in the HIES and thymoma patient with autoantibodies (Fig 2C). No significant differences were found in other cytokines across the groups (S2 Fig). IL-12/18 activation of MAIT cells did not induce cytokine production from 12 out of these 25 cytokines.

MAIT cells are tissue resident innate T cells that provide rapid cytokine production and have been shown to be important in various infectious diseases [16, 23, 24]. Study by Wilson et al. has found that IL-23 is one of the main cytokines responsible for maintaining the number of innate T cells i.e. MAIT cells and iNKT cells. [25] Our findings suggest that there is an additional impairment of MAIT cells in immunodeficiency disorders associated with impaired systematic Th17 immunity. During the review process of this paper Wilson et al [25] showed a quantitative deficiency in a larger group of AD-HIES patients with loss of qualitative function in IL17A and F production. Our work supports this observation and extends it to secondary immunodeficiencies such as the IL-23 autoantibody phenocopy of STAT-3 impairment for MAIT cell function. In accordance with this, the clinical course of our some of our thymoma positive patients was complicated by fungal and bacterial infections (Table 1), suggesting that the clinical impact of this systemic and innate T cell deficiency may have functional importance. Further observations on other immunodeficiencies with similar clinical features and/or systemic Th17 deficiencies are required to better understand the role of innate and conventional T cell compartments in the protection against infectious disease. These observations require further evaluation in larger cohorts but our study highlights the importance of evaluating non-conventional T cell populations in different immunodeficiency disorders to better understand their potential functional role and association with characteristic clinical presentations.

**Table 1:** Clinical details of primary and secondary immunodeficiencies associated with Th17 deficiency.

**Primary Immunodeficiency**												
Patient	Gender	Age	Autoantibodies to IL12/23	Autoimmune Disease	Infections	T cells	T cells %	CD4	CD4cells %	CD8	CD8cells %	B cells
HIES 1*STAT3 c*.*1909G>A V637M*	F	30	-	-	Recurrent bacterial ear, chest and sinus infections (childhood onset) Recurrent *Staphylococcal Aureus* skin infections and deep tissue abscess Bronchiectasis, Lung cysts and Pneumatoceles Recurrent oral candidiasis (childhood onset)	1420	79	900	50	380	21	180
HIES 2 *STAT3 c*.*1251A>T R417S*	F	47	-	-	Recurrent bacterial chest infections (Childhood onset) Recurrent *Aspergillus Fumigatis* chest infections Recurrent oral candidiasis (childhood onset) Bronchiectasis	750↓	68	570	52	140↓	12.7	80↓
HIES 3 *STAT3 c*.*1853G>A G618D*	M	43	-	-	Pneumocystis jirovecii pneumonia Recurrent *Staphylococcal Aureus* skin infections (childhood onset) Recurrent bacterial ear, chest and sinus infections (childhood onset) Bronchiectasis and lung cysts	1480	78	860	45	570	30	240
** Secondary Immunodeficiency **												
P1	F	74	-	-	-	1182	70	1003	85	150↓	12	524
P2	F	64	-	Cerebellar Degeneration	-	569 ↓	52	309	54	246	43	132
P3	F	53	-	Myasthenia Gravis	-	558 ↓	70	268↓	48	276	49	112
P4	M	61	-	-	-	1362	91	603	44	610	45	139
P5	M	57	+	-	-	3110	86	1313	42	1465	47	844
P6	F	62	+	Myasthenia Gravis	Recurrent bacterial respiratory infections Bronchiectasis	922	84	248↓	27	625	68	113
P7	F	65	+	Myasthenia Gravis Crohns Disease	-	1341	71	487	36	431	32	241
P8	M	66	+	Autoimmune enteropathy	Oesophageal Candidiasis	1847	80	1103	60	613	33	225
P9	F	73	+	-	Recurrent bacterial respiratory infections Recurrent *Herpes Simplex* keratitis	1663	79	531	32	1089	66	201
P10	F	64	+	-	-	243↓	35	97↓	40	139↓	57	50↓

## Supporting Information

S1 Fig Proliferation of MAIT cells by anti-CD3/CD28 and IL-12/18. (PDF)

S2 Fig Cytokine production by enriched MAIT cells following IL-12/IL-18 stimulation. (PDF)

S3 Fig IFN-gamma production by MAIT cells following IL-12/IL-18 stimulation. (PDF)
