# Supplementary figures and images for: Mucosal-Associated Invariant T (MAIT) Cells Are Impaired in Th17 Associated Primary and Secondary Immunodeficiencies
Source: PLoS One. 2016 May 11;11(5):e0155059. doi: 10.1371/journal.pone.0155059 (PMC4864289; doi:10.1371/journal.pone.0155059)

Supplementary Figure 1

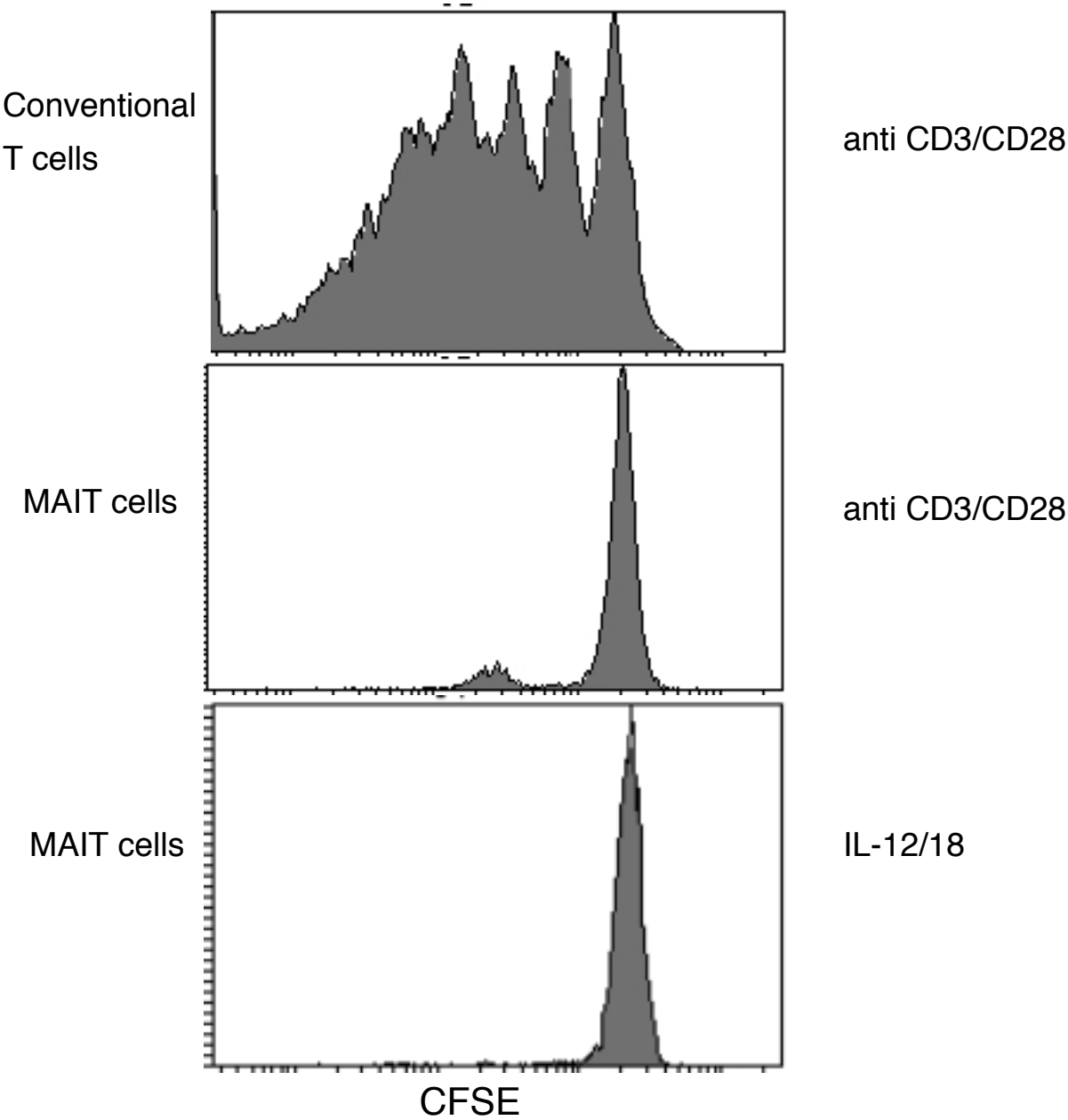

Supplement: S1 Fig — (PDF) [file pone.0155059.s001.pdf]

Supplementary Figure 2

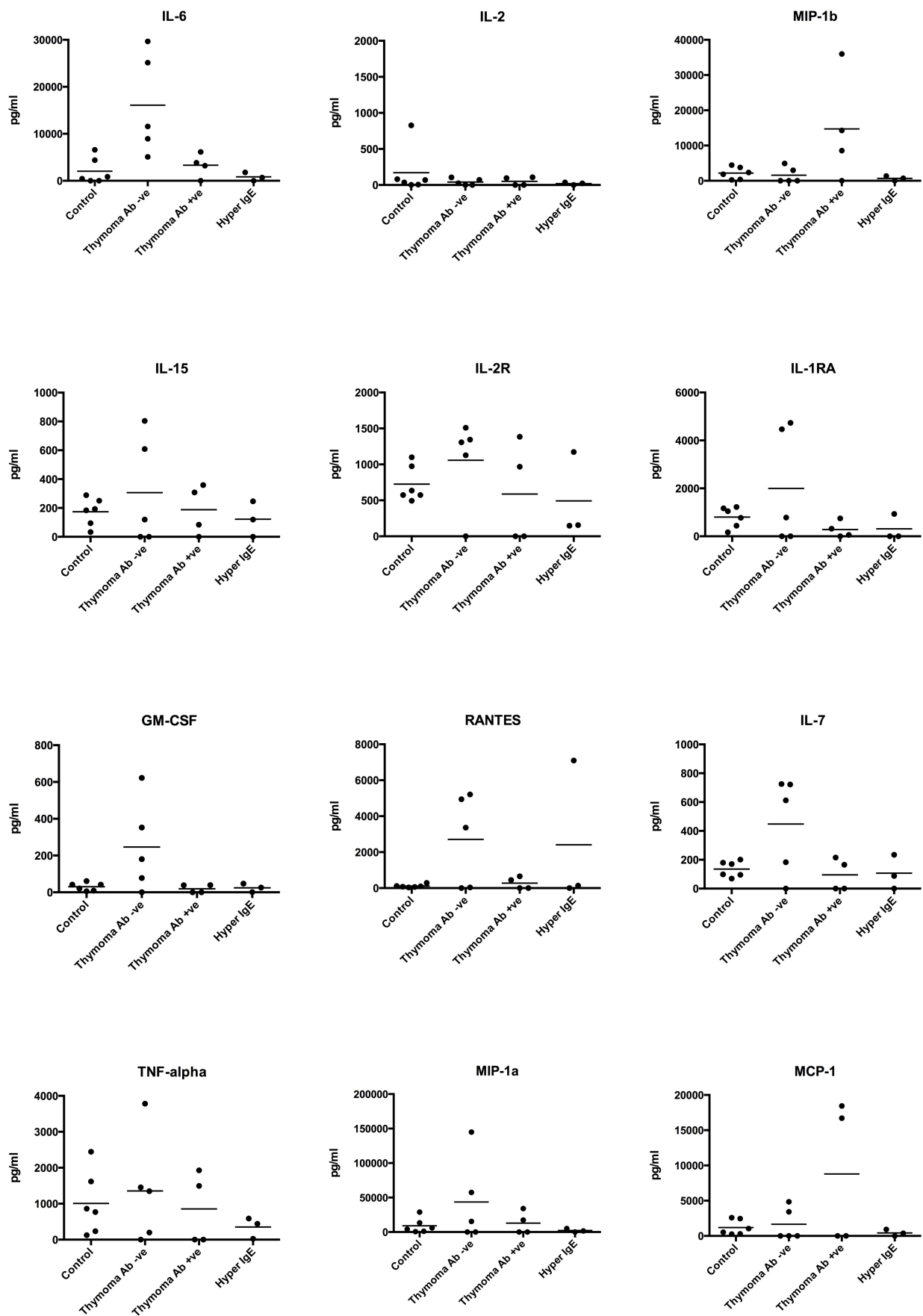

Supplement: S2 Fig — (PDF) [file pone.0155059.s002.pdf]

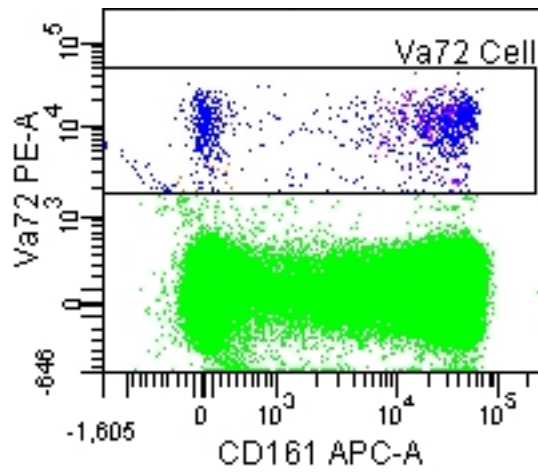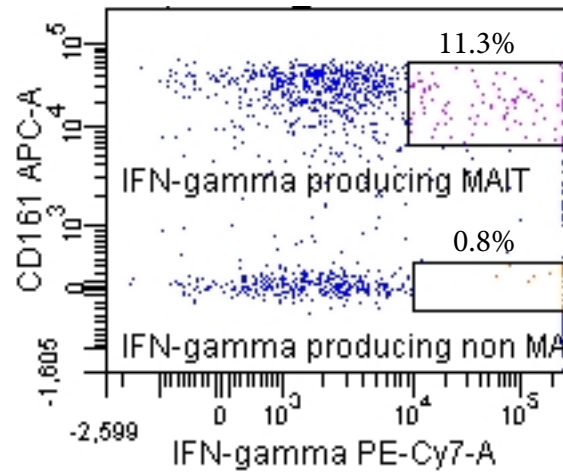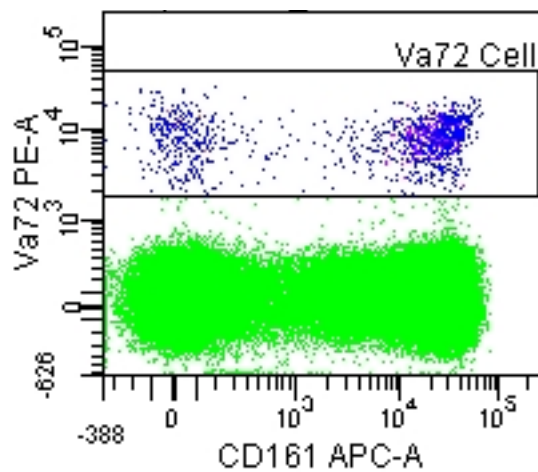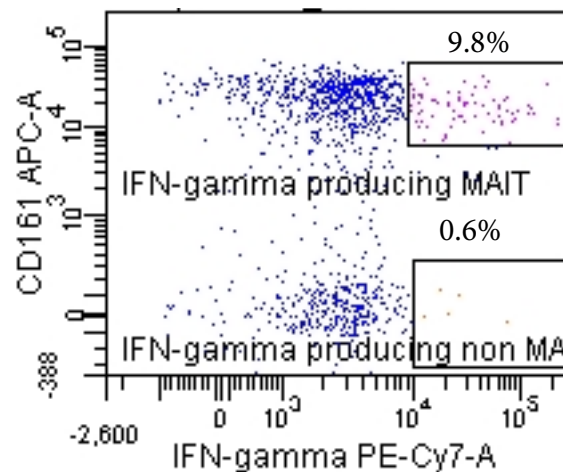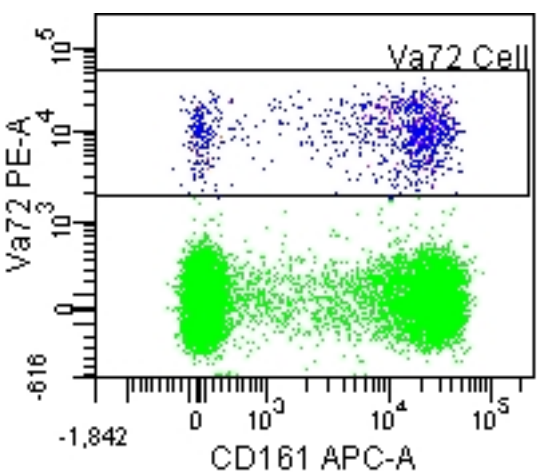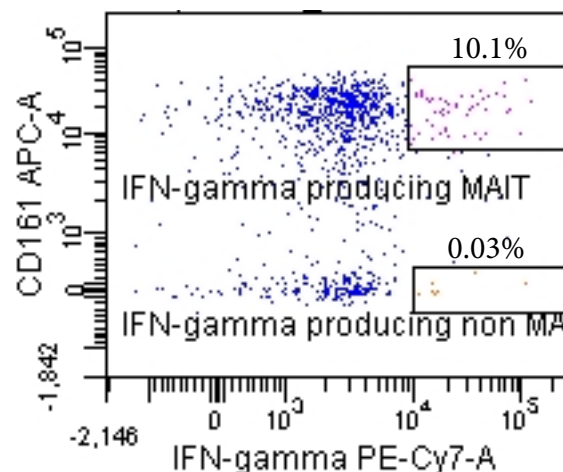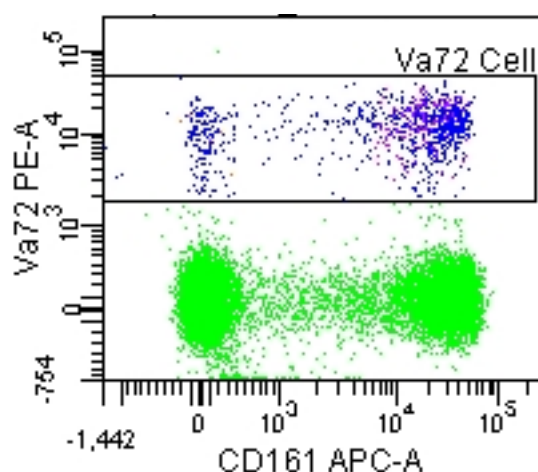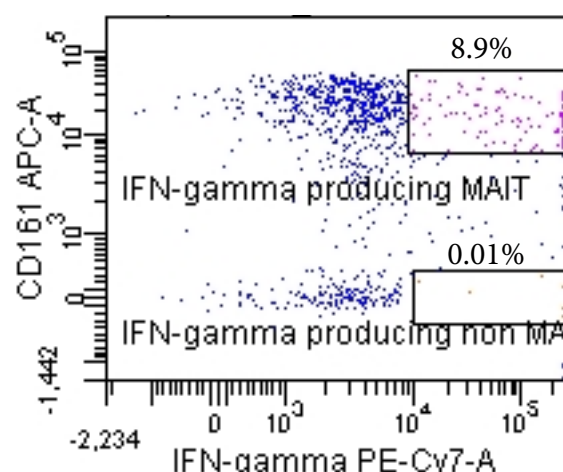

Supplement: S3 Fig — (PDF) [file pone.0155059.s003.pdf]
